# GPER activation attenuates cardiac dysfunction by upregulating the SIRT1/3-AMPK-UCP2 pathway in postmenopausal diabetic rats

**DOI:** 10.1371/journal.pone.0293630

**Published:** 2023-12-22

**Authors:** Hossein Azizian, Zeinab Farhadi, Michael Bader, Jalil Alizadeh Ghalenoei, Mohammad Amin Ghafari, Shokoufeh Mahmoodzadeh

**Affiliations:** 1 Yazd Neuroendocrine Research Center, School of Medicine, Shahid Sadoughi University of Medical Sciences and Health Services, Yazd, Iran; 2 Max-Delbrück-Center for Molecular Medicine in the Helmholtz Association (MDC), Berlin, Germany; 3 DZHK (German Centre for Cardiovascular Research), Partner Site Berlin, Berlin, Germany; 4 Charité Universitätsmedizin Berlin, Corporate Member of Freie Universität Berlin and Humboldt‐Universität zu Berlin, Berlin, Germany; 5 University of Lübeck, Institute for Biology, Lübeck, Germany; Indiana University School of Medicine, UNITED STATES

## Abstract

Postmenopausal diabetic women are at higher risk to develop cardiovascular diseases (CVD) compared with nondiabetic women. Alterations in cardiac cellular metabolism caused by changes in sirtuins are one of the main causes of CVD in postmenopausal diabetic women. Several studies have demonstrated the beneficial actions of the G protein-coupled estrogen receptor (GPER) in postmenopausal diabetic CVD. However, the molecular mechanisms by which GPER has a cardioprotective effect are still not well understood. In this study, we used an ovariectomized (OVX) type-two diabetic (T2D) rat model induced by high-fat diet/streptozotocin to investigate the effect of G-1 (GPER-agonist) on sirtuins, and their downstream pathways involved in regulation of cardiac metabolism and function. Animals were divided into five groups: Sham-Control, T2D, OVX+T2D, OVX+T2D+Vehicle, and OVX+T2D+G-1. G-1 was administrated for six weeks. At the end, hemodynamic factors were measured, and protein levels of sirtuins, AMP-activated protein kinase (AMPK), and uncoupling protein 2 (UCP2) were determined by Western blot analysis. In addition, cardiac levels of oxidative stress biomarkers were measured. The findings showed that T2D led to left ventricular dysfunction and signs of oxidative stress in the myocardium, which were accompanied by decreased protein levels of Sirt1/2/3/6, p-AMPK, and UCP2 in the heart. Moreover, the induction of the menopausal state exacerbated these changes. In contrast, treatment with G-1 ameliorated the hemodynamic changes associated with ovariectomy by increasing Sirt1/3, p-AMPK, UCP2, and improving oxidative status. The results provide evidence of the cardioprotective effects of GPER operating through Sirt1/3, p-AMPK, and UCP2, thereby improving cardiac function. Our results suggest that increasing Sirt1/3 levels may offer new therapeutic approaches for postmenopausal diabetic CVD.

## Introduction

Type 2 diabetes (T2D), in particular in women, is an emerging epidemic and causes thousands of deaths worldwide each year, directly and indirectly, due to comorbidities, including cardiovascular diseases (CVD) [1]. Also, Menopause is associated with an increase in metabolic disorders such as T2D, leading to changes in mitochondrial function and cardiac metabolism, resulting in left ventricular hypertrophy and extracellular fibrosis [2]. Diabetes induced hyperglycemia affects heart structure and function by altering insulin signaling, oxidative stress, mitochondrial function, impaired calcium handling, increased inflammation, and increased consumption of fatty acids as energy sources. Since the heart works constantly and intensively, it requires a large amount of energy in the form of ATP molecules from the mitochondria. Therefore, cardiac mitochondria play a crucial role in the proper functioning of the heart [3]. Since mitochondrial dysfunction and oxidative stress play an essential role in diabetes-related CVD, targeting mitochondria may lead to effective treatment of diabetic-induced CVD [4]. One of the mechanisms of impaired physiological function of mitochondria is post-translational changes in mitochondrial proteins, which are mainly mediated by acetylation [5]. Sirtuins (Sirt) are a family of class III histone deacetylases (HDACs) that are different from other classes of HDACs due to the need for the nicotinamide adenine dinucleotide (NAD), which modulate the acetylation status of proteins. Sirtuins’ need for NAD probably indicates their involvement in regulating the expression of genes affecting cardiac metabolism and nutritional status [4]. Among the seven sirtuins found in mammals, Sirt1, Sirt2, Sirt3, and Sirt6 play a critical role in regulating cellular metabolism and mitochondrial function by modulating the acetylation of histones and non-histone proteins. Thus, these sirtuins also regulate the transcription of several essential genes involved in cardiac metabolism [5].

AMP-activated protein kinase (AMPK) is a critical cellular sensor of energy availability that balances oxidative stress, and under conditions such as obesity and diabetes, phosphorylation and consequently its activity decreases [6]. The AMPK signaling pathway is a major regulator of mitochondrial function [7], and it has been suggested that AMPK may also activate sirtuins by increasing NAD+ levels [8]. Impairment of the AMPK signaling pathway is also a contributing factor to diseases caused by diabetes and metabolic syndromes such as cardiomyopathy and nephropathy [9]. In addition, AMPK has been shown to have pleiotropic cardioprotective effects that restore cardiac dysfunction by improving energy supply and regulating other physiological processes [6]. Uncoupling proteins (UCPs), located in the inner mitochondrial membrane, participate in maintaining mitochondrial function and regulating oxidative stress [10]. UCP2, a member of the UCP family, can protect the cardiomyocytes against oxidative stress by inhibiting reactive oxygen species (ROS) generation [10]. In addition, AMPK regulates the activity of UCP2 [11] and has pleiotropic cardioprotective effects by improving energy supply and regulating other physiological processes [6].

17β-estradiol (E2), the main female estrogen, exerts protective effects on energy status and cardiac metabolism through various molecular and cellular pathways [12]. Thus, postmenopausal women show more susceptibility to metabolic disorders caused by E2 reduction [13]. The protective metabolic effects of E2 are traditionally attributed to classical E2 receptors, which are mediated by genomic pathways, a time-consuming and slow process [14]. Recently, increasing evidence has demonstrated that the G protein-coupled estrogen receptor (GPER), as a 7-transmembrane receptor is expressed in a wide variety of tissues such as the heart and blood vessels, and mediates the rapid biological action of E2 [15]. Based on genetic and pharmacological approaches, there is a growing body of literature emphasizing the role of GPER in the regulation of metabolic function of E2 as well as the regulation of cardiac function [12]. Recently, we and other authors demonstrated that E2 via GPER has metabolic cardiovascular and anti-inflammatory protective effects in postmenopause, T2D, and obesity [12, 15]. However, the detailed mechanisms by which GPER can improve metabolic and cardiovascular disorders are not yet fully characterized [12].

Thus, in light of the above, the aim of the present study was to investigate whether GPER regulates the cardiac sirtuins in postmenopausal T2D rats. To test this hypothesis, we modeled menopausal T2D condition (a menopausal diabetic state) and GPER agonist treatment in female rats.

## Methods

### Chemicals and antibodies

Streptozotocin (STZ) and Dimethyl sulfoxide (DMSO), and saline were gained from Sigma-Aldrich (USA), while G-1 (selective GPER-agonist) was gained from Tocris Bioscience (USA). STZ was dissolved in 0.1 M citrate buffer (pH 4.4), while G-1 was dissolved in 16% DMSO in 0.9% saline and was used as a vehicle for G-1 [16]. Radio immunoprecipitation assay (RIPA) lysis buffer, the BCA protein assay kit, and the enhanced chemiluminescence (ECL) plus kit were obtained from Santa Cruz Biotechnology, Pierce (USA), and Amersham (Arlington Heights, IL), respectively. Primary antibodies used for Western blot analyses were as follows: anti-SIRT1, anti-SIRT2, anti-SIRT3, anti-SIRT6, and anti-UCP2 were obtained from Santa Cruz Biotechnology (Texas, USA), and Phospho-AMPK (Thr172), AMPK, and β-actin antibody were obtained from Cell Signaling Technology (INC. Beverly, MA, USA). Secondary antibodies were obtained from Santa Cruz Biotechnology (Texas, USA).

### Animals and experimental design

**Fig 1:**
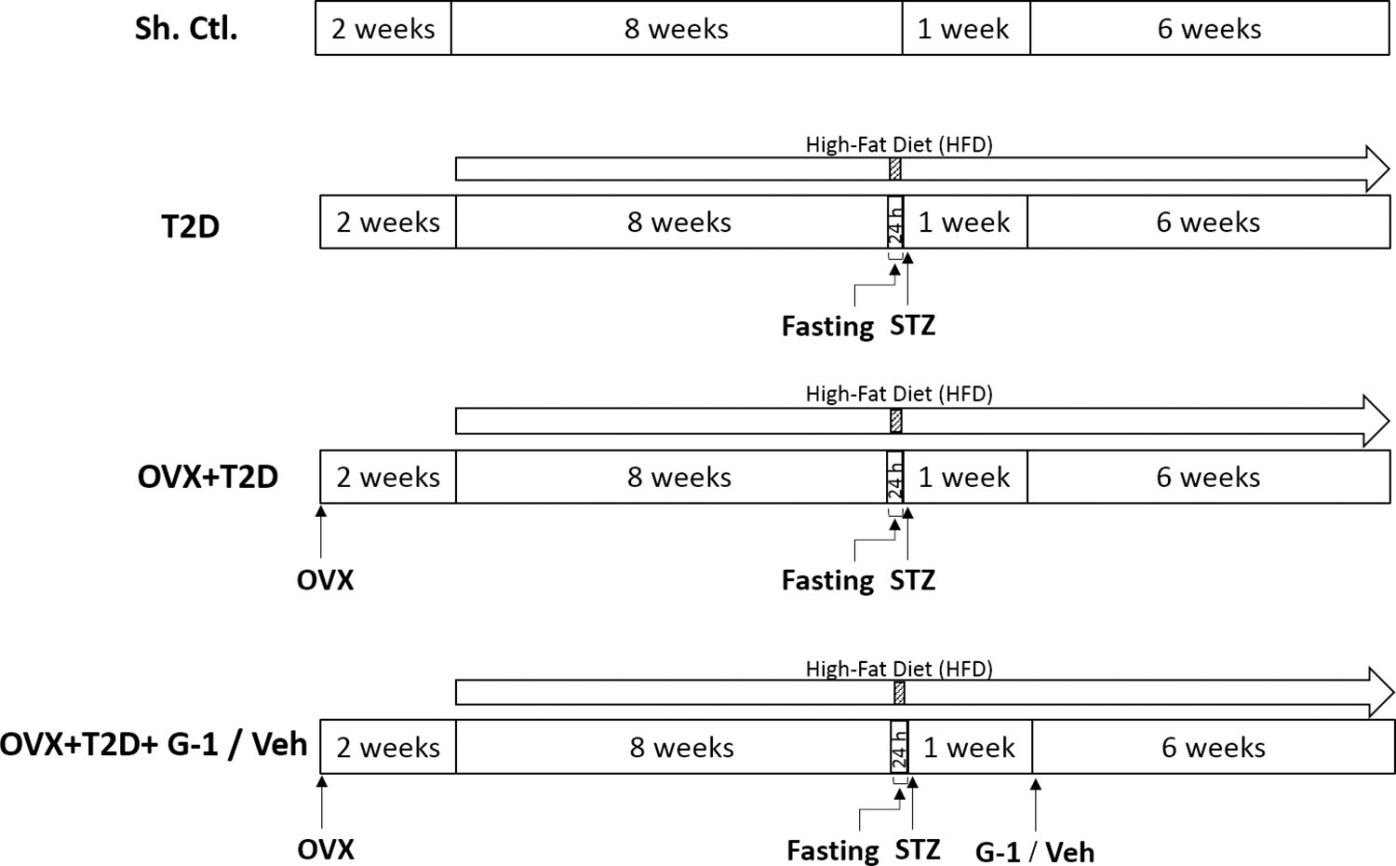
Schematic of the experimental design. G-1: GPER agonist, h: Hours, OVX: Ovariectomized, Sh-Ctl: Sham-Control, STZ: Streptozotocin, T2D: Type 2 diabetes, Veh: Vehicle.

Female Wistar rats (3–4 months, 210–230 g) were obtained from the Animal Center of Shahid Sadoughi University of Medical Sciences, Yazd, Iran. Female rats were maintained in an air-conditioned (22–25°C) room, light-dark cycle (12:12 h), and free access to food and water. This study was in accordance with the ethical guidelines of the National Institutes of Health on the care and use of animals and was approved by the Institutional Animal Care Committee of Shahid Sadoughi University (IR.SSU.1400.285). Female rats were randomly assigned to five groups (7–10 rats in each group): Sham-Control (Sh-Ctl); T2D; Ovariectomized (OVX)+T2D; OVX+T2D+Vehicle (Veh); and OVX+T2D+G-1. Animals were OVX two weeks before the initiation of experiments as previously described [15]. To induce the T2D model, female rats were given a high-fat diet (HFD) for 8 weeks. Subsequently, the animals were fasted for 24 hours and received an i.p. injection of STZ (30 mg/kg). Fasting glucose levels were confirmed to be over 200 mg/dl in rats one week after injection [17]. After confirmation of T2D, female rats were given i.p. injections of G-1 (200 μg/Kg) or vehicle three days per week for 6 weeks [12]. The percentage of body weight (BW) changes was calculated as (final BW–initial BW) divided by initial BW times 100, where initial BW was BW at Day 1 of treatment. A timeline of this experimental process is shown in Fig 1.

### Hemodynamic and metabolic parameters

Following the completion of the treatment protocol, the female rats were deeply anesthetized with an i.p injection of a mixture of ketamine/xylazine (80/10 mg/kg). After making an incision in the neck region, a cannula was placed in the trachea of the animal to ventilate in the case of emergency. Then a polyethylene catheter (PE-50) filled with heparin saline was placed in the right carotid artery, and advanced to the left ventricle. The mean arterial pressure (MAP) and hemodynamic indices including left ventricular systolic pressure (LVSP), left ventricular end-diastolic pressure (LVEDP), the maximum rate of rise of left ventricular pressure during systole (+dp/dt max), and the maximum rate of reduction in pressure during diastole (−dp/dt max) were recorded on an 8-channel PowerLab Physiograph system (ADInstruments, Australia) [18].

Following the completion of the hemodynamic experiments, the female rats were deeply anesthetized with an i.p injection of a mixture of ketamine/xylazine. The hearts were rapidly removed, rinsed in phosphate-buffered saline (PBS), weighed, and then frozen in liquid nitrogen. Fasting blood glucose (FBG) levels were measured using hexokinase method on fully automated chemistry analyzer Olympus AU400 (Olympus Corporation, Japan) [19].

### Estimation of malondialdehyde (MDA) and superoxide dismutase (SOD) in cardiac tissue

For each rat, a portion of the left ventricle was homogenized in ice-cold lysis buffer and then centrifuged at 11,269 x g and 4°C for 10 min. The supernatant was kept at −80°C for further processing steps. The SOD activity and MDA level, a lipid peroxidation product, were determined using commercially available ELISA kits (ZellBio GmbH, Germany) according to the manufacturer’s instructions.

### Sample preparation and Western blotting

The left ventricle from each rat was homogenized in ice-cold lysis buffer and centrifuged at 15,339 x g and 4°C for 15 min. The protein concentrations were measured using the BCA method. Equal amounts of protein were subjected to SDS-PAGE gel and the separated proteins transferred onto nitrocellulose membrane. The membrane was blocked (overnight at 4°C) with 5% skimmed milk and then incubated with primary antibodies against Sirt1, Sirt2, Sirt3, Sirt6, UCP2, p-AMPK (diluted 1:1000) and β-actin (diluted 1:2000), respectively, for three hours at room temperature. After washing in phosphate buffered saline with tween-20 (1x PBST) (three times, 5 min each), the membrane was incubated with a horseradish peroxidase-conjugated secondary antibody (1:15,000) for 60 min at room temperature. The protein band density was detected using the enhanced chemiluminescence (ECL) system, and Image J software (UVP, UK) was used to quantify band intensities relative to the loading control β-actin or AMPK expression.

### Statistical analyses

Data were expressed as mean ± standard error of the mean (±SEM). Statistical analyses were performed using GraphPad Prism (Version 6.0) software. Statistical differences were assessed using one-way analysis of variance, followed by Tukey-Kramer test. P ≤ 0.05 was considered statistically significant.

## Results

### GPER activation counteracts the effects of menopause on BW, FBG, and HW

In current study, as shown in Table 1, induction of T2D in female rats led to a significant increase in FBG (P ≤ 0.001), BW, and HW (P ≤ 0.01) compared with the Sh-Ctl group. All three parameters—BW (P ≤ 0.01), FBG, and HW (P ≤ 0.05)—were further significantly increased by inducing the menopausal state in T2D rats (OVX+T2D vs. T2D). However, G-1 treatment significantly inhibited the elevation of BW, FBG, and HW (P ≤ 0.05) in OVX-T2D female rats. However, since no statistically significant differences were found between the OVX+T2D+vehicle and OVX+T2D groups for any of the other parameters measured, we refrain to mention this hereafter for the other parameters.

**Table 1:** Effects of GPER activation on body weight, fasting blood glucose, and heart weight in OVX-T2D female rats.

Groups	Sh-Ctl	T2D	OVX+T2D	OVX+T2D+Veh	OVX+T2D+G-1
**% BW Change**	8.6 ± 0.5	11.9 ± 0.8**	15.4 ± 0.7^##^	16.2 ± 0.5	13.6 ± 0.3^+^
**FBG (mmol/L)**	4.5 ± 0.2	16 ± 1.8***	21.3 ± 0.2^#^	22.1 ± 2.3	19.5 ± 0.3^+^
**HW (mg)**	541 ± 50	748 ± 49**	918 ± 43^#^	993 ± 26	828 ± 23^+^

Data are presented as mean±SEM.

** P ≤ 0.01 and

*** P ≤ 0.001, vs. Sh-Ctl.

^#^ P ≤ 0.05 and

^##^ P ≤ 0.01 vs. T2D.

^+^ P ≤ 0.05 vs. OVX+T2D+Veh. BW: Body weight, FBG: Fasting blood glucose, G-1: GPER agonist, HW: Heart weight, OVX: Ovariectomized, Sh-Ctl: Sham-control, T2D: Type 2 diabetes, Veh: Vehicle.

### GPER activation improves cardiac function in OVX-T2D female rats

**Fig 2:**
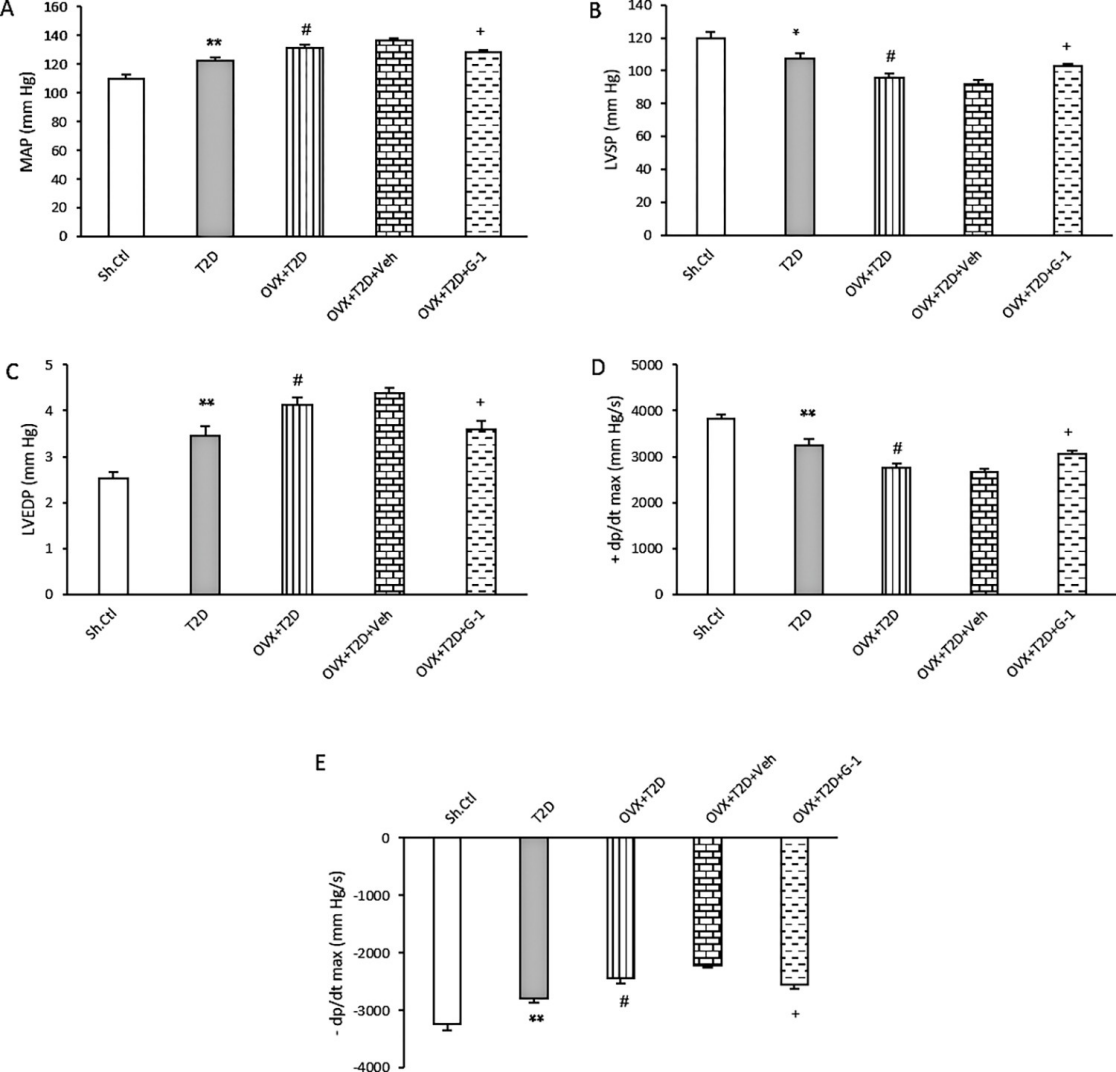
Basal hemodynamic and left ventricular indices in experimental groups. (A) MAP, (B) LVSP, (C) LVEDP, (D) +dp/dt max, (E) −dp/dt max. All data are expressed as mean±SEM. * P ≤ 0.05 and ** P ≤ 0.01 vs. Sh-Ctl. ^#^ P ≤ 0.05 vs. T2D. ^+^ P ≤ 0.05 vs. OVX+T2D+Veh. One-way ANOVA followed by Tukey-Kramer test. +dp/dt max: Maximum rate of increase in left ventricular pressure during systole, −dp/dt max: Maximum rate of decrease in left ventricular pressure during diastole, G-1: GPER agonist, LVEDP: Left ventricular end diastolic pressure, LVSP: Left ventricular systolic pressure, MAP: Mean arterial pressure, OVX: Ovariectomized, Sh-Ctl: Sham-control, T2D: Type 2 diabetes, Veh: Vehicle.

Fig 2 shows changes in basal hemodynamic and left ventricular indices in different groups. As expected, induction of T2D resulted in a significant increase in MAP and LVEDP (P ≤ 0.01), and a significant decrease in ± dp/dt (P ≤ 0.01) and LVSP (P ≤ 0.05) compared with the Sh-Ctl group. However, the induction of menopausal states in T2D animals enhanced the observed changes. Thereby, menopause together with diabetes (OVX+T2D) caused significantly more increased MAP and LVEDP levels and significantly greater reduction in LVSP and ± dp/dt compared with the T2D group (P ≤ 0.05). Interestingly, our data showed that activation of GPER by G-1 markedly affected cardiac function in OVX+T2D rats. Treatment with G-1 led to a significant decrease in MAP and LVEDP and a significant increase in LVSP and ± dp/dt in OVX-T2D animals in comparison with the vehicle group (P ≤ 0.05).

### GPER mediates the upregulation of Sirt1 and Sirt3 expression in the left ventricle of OVX-T2D female rats

**Fig 3:**
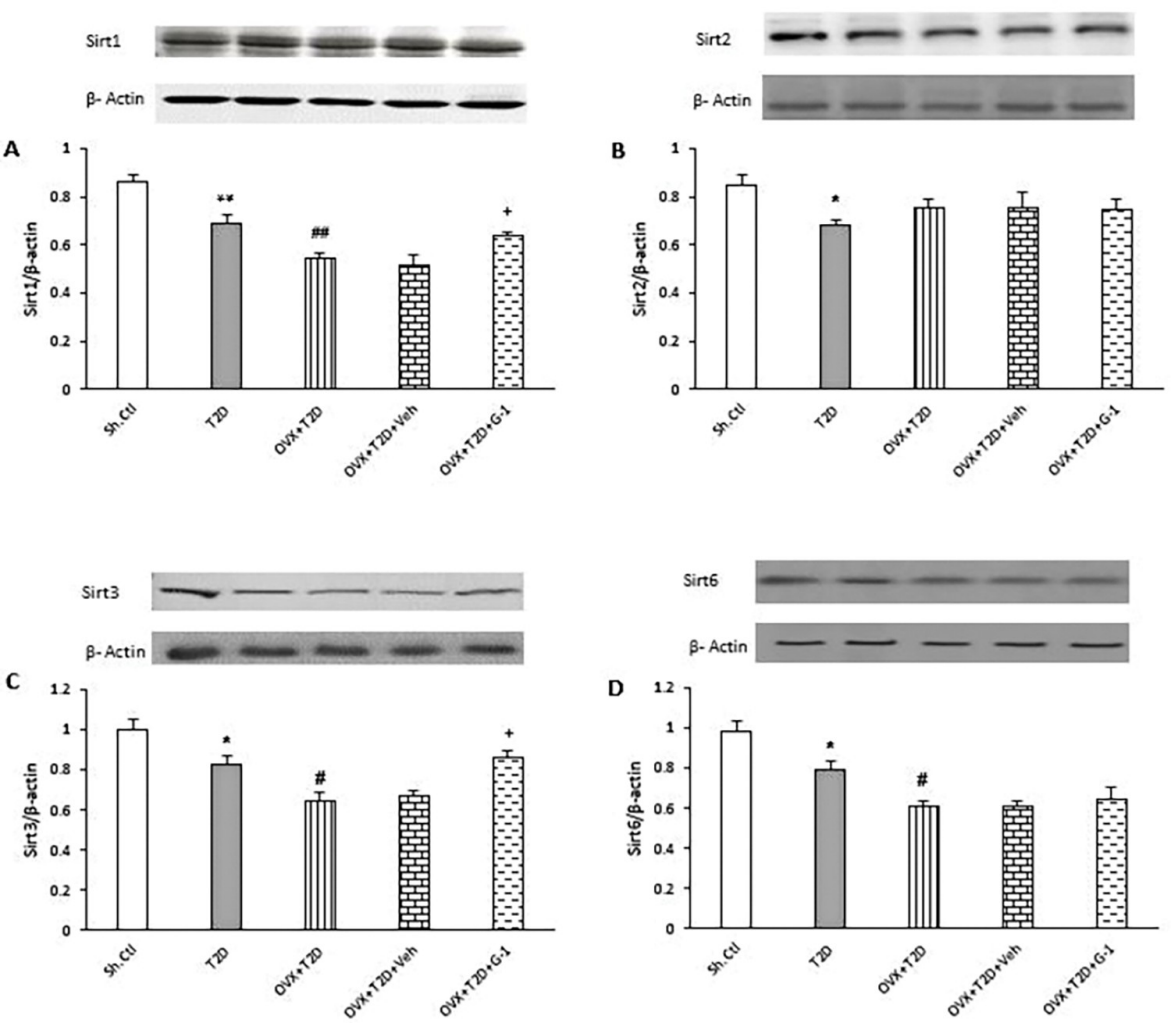
Western blot analysis of (A) Sirt1, (B) Sirt2, (C) Sirt3, and (D) Sirt6 protein levels in the left ventricle of each group. In each graph: Upper panels: Representative immunoblots for the respective Sirt proteins (as indicated) and β-actin (as internal control) in the left ventricle tissues of female rats of the examined groups. Lower panels: Expression of respective Sirt proteins as the relative band intensity to actin band intensity ratio in each group. All data are expressed as mean±SEM. * P ≤ 0.05 and ** P ≤ 0.01 vs. Sh-Ctl. ^#^ P ≤ 0.05 and ^##^ P ≤ 0.01 vs. T2D. ^+^ P ≤ 0.05 vs OVX+T2D+Veh. One-way ANOVA followed by Tukey-Kramer test. G-1: GPER agonist, OVX: Ovariectomized, Sh-Ctl: Sham-control, Sirt1: Sirtuin 1, Sirt2: Sirtuin 2, Sirt3: Sirtuin 3, Sirt6: Sirtuin 6, T2D: Type 2 diabetes, Veh: Vehicle.

Several studies reported that the level of sirtuin proteins are affected in diabetic hearts [5, 20]. In line with these data, as shown in Fig 3, we could show that the induction of T2D in female rats significantly reduced the cardiac levels of Sirt1 (P ≤ 0.01), Sirt2, Sirt3, and Sirt6 proteins (P ≤ 0.05) compared with the Sh-Ctl group. Induction of ovariectomy in T2D rats caused a greater reduction in the cardiac levels of Sirt1 (P ≤ 0.01), Sirt3, and Sirt6 (P ≤ 0.05) expression compared with the T2D group. Intriguingly, activation of GPER with G-1 markedly reversed the reduction in protein expression of Sirt1 and Sirt3 induced by menopause (P ≤ 0.05). Neither ovariectomy nor the GPER agonist G-1 had any effect on the level of Sirt2 protein expression.

### GPER agonist G-1 promotes the phosphorylation of AMPK in the left ventricle of OVX-T2D female rats

**Fig 4:**
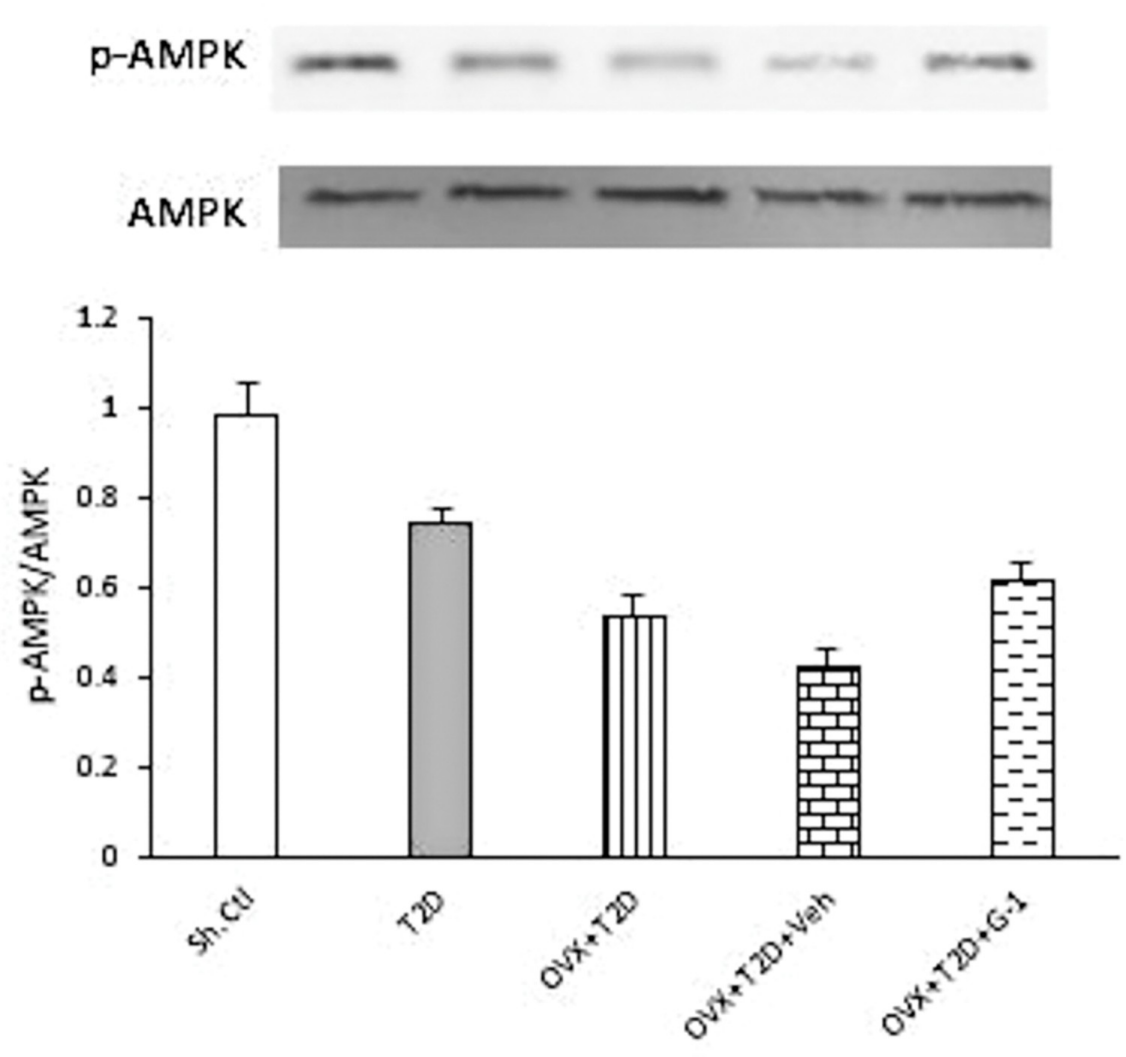
Western blot analysis of p-AMPK expression levels in the left ventricle of each group examined. Upper panel: Representative immunoblot for p-AMPK and AMPK in the left ventricle tissues of female rats of the examined groups. Lower panel: Expression of p-AMPK as the relative band intensity to AMPK band intensity ratio in each group. All data are expressed as mean±SEM. ** P≤0.01 vs. Sh-Ctl. ^#^ P ≤ 0.05 vs. T2D. ^+^ P ≤ 0.05 vs OVX+T2D+Veh. One-way ANOVA followed by Tukey-Kramer test. G-1: GPER agonist, OVX: Ovariectomized, Sh-Ctl: Sham-control, T2D: Type 2 diabetes, Veh: Vehicle.

Since AMPK is a key regulator of energy metabolism in the heart, we investigated in our study whether cardiac AMPK activation (i.e., phosphorylation of AMPK (p-AMPK)), is altered in different animal groups. As shown in Fig 4, induction of T2D in female animals significantly reduced the cardiac level of p-AMPK protein compared to Sh-Ctl group (P ≤ 0.01). While ovariectomy significantly enhanced the reduction of p-AMPK protein levels in the left ventricle of female T2D animals (OVX+T2D vs. T2D, P ≤ 0.05), treatment with the GPER agonist G-1 significantly reversed the attenuation of p-AMPK levels in the left ventricle of these animals (OVX+T2D+G-1 vs. OVX+T2D+Veh, P ≤ 0.05).

### GPER activation increased the level of UCP2 and reduced oxidative stress in the left ventricle of OVX-T2D female rats

**Fig 5:**
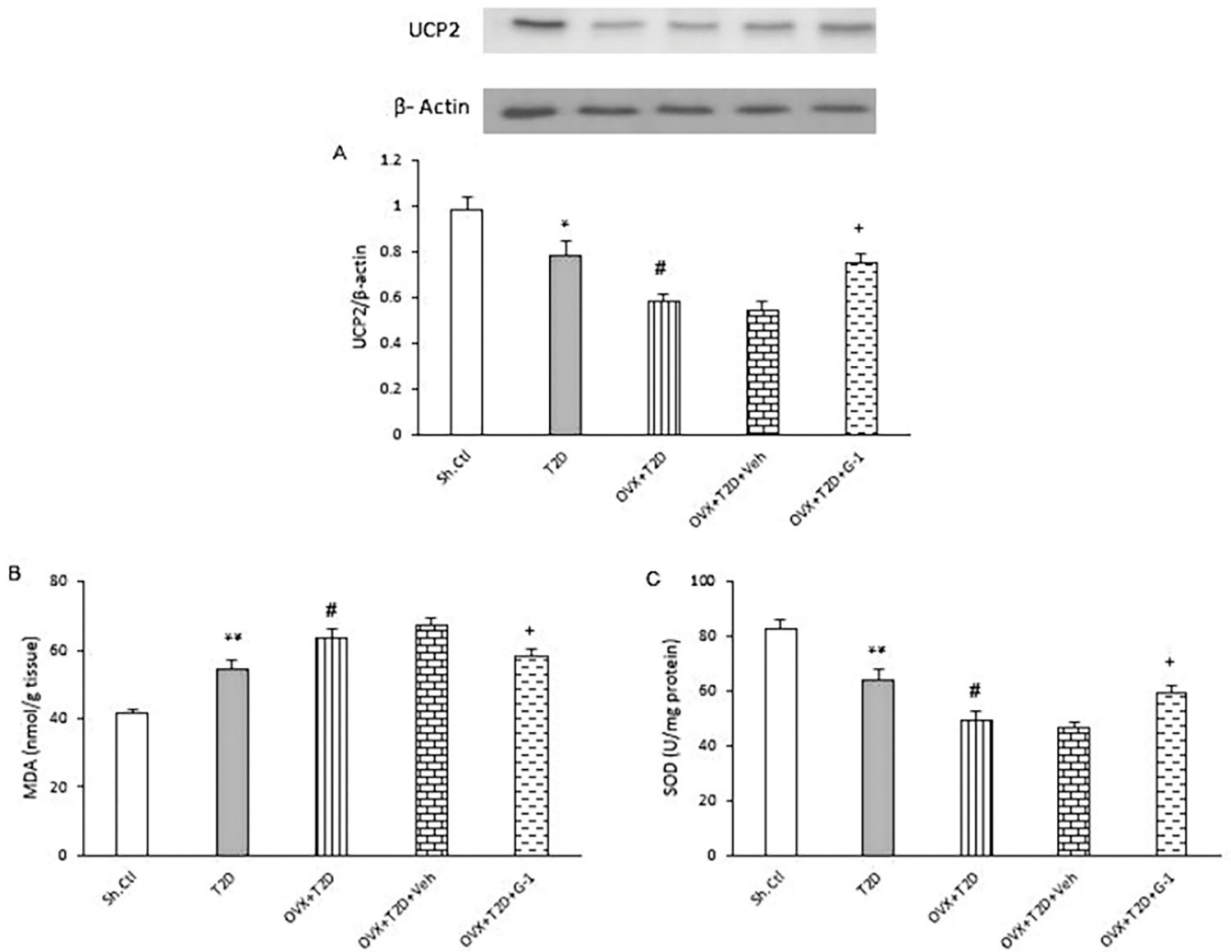
(A) Western blot analysis of UCP2 protein expression levels and (B, C) measurements of MDA and SOD levels (by ELISA) in the left ventricle of each group examined. (A) Upper panel: Representative immunoblot for UCP2 and β-actin (as internal control) in the left ventricle tissues of female rats of the examined groups. Lower panel: Expression of UCP2 as the relative band intensity to β-actin band intensity ratio in each group. Changes of MDA contents (B) and SOD activity (C) in the left ventricle of each group. All data are expressed as mean±SEM. ** P ≤ 0.01 vs. Sh-Ctl. ^#^ P ≤ 0.05 vs. T2D. ^+^ P ≤ 0.05 vs OVX+T2D+Veh. One-way ANOVA followed by Tukey-Kramer test. G-1: GPER agonist, MDA: Malondialdehyde, OVX: Ovariectomized, Sh-Ctl: Sham-control, SOD: Superoxide dismutase, T2D: Type 2 diabetes, UCP2: Uncoupling protein 2, Veh: Vehicle.

Next, we investigated the changes in the expression of UCP2 protein, which is primarily expressed in the inner mitochondrial membrane and regulates mitochondrial biogenesis, reactive oxygen species (ROS) production, and fatty acid oxidation. As shown in Fig 5A, the expression of UCP2 protein was significantly decreased in the left ventricle of T2D female rats in comparison to Sh-Ctl group (T2D vs. Sh-Ctl, P ≤ 0.05). Again, the induction of ovariectomy state in these rats led to further significant reduction in the level of UCP2 protein expression (OVX+T2D vs. T2D, P ≤ 0.05). However, treatment with a GPER agonist (G-1) reduced the effects of ovariectomy and was associated with upregulation of UCP2 protein levels in the left ventricle of these rats (OVX+T2D+G-1 vs. OVX+T2D+Veh, P ≤ 0.05).

Finally, to clarify the effect of GPER activation on oxidative stress and anti-oxidative defense, the changes in the left ventricle levels of MDA and SOD were examined (Fig 5B and 5C). Cardiac MDA level was increased significantly in T2D group as compared with Sh-Ctl group (P ≤ 0.01, Fig 5B). Induction of ovariectomy in female T2D rats was associated with significantly higher cardiac MDA levels than in female T2D rats (P ≤ 0.05, Fig 5B). In contrast, treatment with GPER agonist (G-1) caused a significant decrement in cardiac level of MDA (OVX+T2D+G-1 vs. OVX+T2D+Veh, P ≤ 0.05). Cardiac activity of SOD showed a significant decline in T2D animals as compared with Sh-Ctl group (P ≤ 0.01, Fig 5C). Ovariectomy led to a more pronounced reduction of SOD activity in the left ventricle of T2D female rats (OVX+T2D vs. T2D, P ≤ 0.05). However, treatment with the GPER agonist (G-1) counteracted these changes, rising the cardiac SOD activity (OVX+T2D+G-1 vs. OVX+T2D+Veh, P ≤ 0.05, Fig 5C).

## Discussion

Cardiometabolic disorders are exacerbated by E2 deficiency and T2D and when both are combined, cardiometabolic disorders becomes accelerated, and additive CVD occurs [21]. Currently, the mechanisms involved in the development of cardiometabolic disorders due to diabetes in postmenopausal women remain unclear, and effective treatment options are limited [21, 22]. Several studies considered mitochondria, especially mitochondrial sirtuins, as a promising target for the management of cardiometabolic disorders [5, 21, 23]. The existing animal models have provided the appropriate conditions for a more detailed investigation of the mechanisms of cardiometabolic disorders, and the present study was conducted to address the effects of the GPER on the regulation of cardiac mitochondrial function in postmenopausal T2D rats. Using this animal model, we found out: First, downregulation of cardiac sirtuins, p-AMPK and UCP2 levels by T2D may increase cardiac production of MDA and decrease SOD activity, and consequently cause cardiac dysfunction. Second, induction of menopausal state (ovariectomy) aggravated the cardiac dysfunction in diabetic hearts through a pronounced reduction of Sirt1/3/6, p-AMPK, and UCP2 dependent mechanism. Third, chronic administration of G-1 increased the cardiac levels of Sirt1/3, p-AMPK, and UCP2 expression, and ameliorated cardiac oxidative stress and partially restored cardiac dysfunctions induced by menopause in ovariectomized T2D female rats.

Maintaining a stable weight depends on the balance between energy intake and expenditure. One possible mechanism that can increase energy expenditure is increased mitochondrial UCPs [12]. Diabetes [24] and ovariectomy [25] can lead to weight gain due to increased energy intake. Molecular studies have revealed that the expression of UCPs was increased in female mice treated with G-1, which can counteract obesity [12]. Consistent with these data, we also observed in our study that female rats with T2D and OVX had significantly increased body weight gain, and treatment with G1 revised these effects, which was most likely related to the increase in UCP2 expression in these rats.

Several studies reported the detrimental effect of T2D on cardiac structure and function [26, 27]. Consistent with these data, we also showed in our study that diabetic female rats exhibited cardiac hypertrophy and increased MAP and LVEDP, as well as decreased LVSP and ± dp/dt compared with the Sh-Ctl group. Clinically it has been shown that the heart of diabetic patients was associated with gene expression changes typical for cardiac dysfunction [28]. These changes in cardiac structure and hemodynamic parameters in T2D condition are attributed to inflammation [29], hyperglycemia, and hyperinsulinemia [30]. In the current study, severe impairment of hemodynamic parameters were observed in OVX-T2D, supporting previous reports that ovariectomy adversely affects cardiac muscle mass [31] and function in diabetic rats [25, 32]. Also, menopause negatively affects left ventricular systolic and diastolic functions in women [33]. Interestingly, in the current study, treatment of OVX+T2D rats with the GPER agonist G-1 resulted in an improvement in both systolic and diastolic function of the heart, as measured by an increase in LVSP, ± dp/dt max, and reduction in LVEDP. It has been reported that chronic GPER activation by its agonist G-1 attenuated the adverse effects of ovariectomy on diastolic function and left ventricle remodelling in female hypertensive rats [34]. In addition, G-1 as a GPER agonist attenuated left ventricular hypertrophy and restored the diastolic function in vivo in rats [35]. GPER activation improves contractile function and reduces infarct size in isolated rat and mouse hearts subjected to ischaemia/reperfusion injury [36, 37]. It has also been shown that a chronic subcutaneous infusion of G-1 inhibited interstitial myocardial fibrosis and improved filling pressures in ovariectomized rats [34].

The results of this study showed that the induction of T2D by the HFD-STZ method reduces the expression of Sirt1, Sirt2, Sirt3, and Sirt6, and when we induced menopause in T2D animals, a further decrease was observed in Sirt1, Sirt3, and Sirt6 levels. However, treatment with G-1 compensated for the decrease in Sirt1 and Sirt3 and increased their expression. Consistent with the results of this study, others have shown that diabetes and obesity reduce the expression of Sirt1 [9], Sirt2 [5], Sirt3 [38], and Sirt6 [39]. Also, Song et al. [40] demonstrated that Sirt1 expression is suppressed in obese and type 2 diabetic women. Moreover, the expression of Sirt1 [41], Sirt3 [42] and Sirt6 [43] decreases in ovariectomy condition. Since Sirt1, Sirt2, Sirt3, and Sirt6 are involved in glucose uptake in the heart, these sirtuins play important roles in regulating metabolism and cardiac function [5, 22]. Additionally, it has been reported that a decrease in Sirt1 and Sirt3 levels in diabetes can cause cardiac complications by acting on mitochondrial proteins [44]. So far, there are very few studies that have investigated the role of GPER on cardiac sirtuins. Resveratrol, a GPER agonist, has been shown to counteract the effects of diabetes by increasing the activity and expression of Sirt1 and Sirt3 in the heart and improving cardiac mitochondrial function [20, 41]. Moreover, it has been shown that activation of estrogen receptors (ERs) increases Sirt1 expression in the heart by activating the AMPK/mammalian target of rapamycin (mTOR) signaling pathway [45, 46]. In addition, studies have shown that activation of ERs increases Sirt3 expression by activating the Sirt3 promoter [47] and increasing nuclear translocation of Nrf2 [48], which in turn reverses mitochondrial dysfunction.

Nowadays, biological processes involved in energy homeostasis, such as the AMPK signaling pathway, have been targeted to counteract insulin resistance and metabolic disorders [49]. In this regard, we found that diabetes reduced p-AMPK in the heart, and while menopause exacerbated this decrease, G-1 was able to counteract it. There is ample evidence that impaired AMPK regulation plays an important role in the development of insulin resistance and T2D, and activation of AMPK, both physiologically and pharmacologically, can prevent these disorders [50–53], and there is also evidence that AMPK activity is reduced in the skeletal muscles of postmenopausal women [54]. It has been shown that induction of menopause in rodents resulted in decreased expression and activity of AMPK in various tissues [55, 56] and E2 therapy reversed these changes within minutes and regulated glucose uptake into muscle, suggesting that the non-genomic pathway of E2 and GPER likely played an important role in this process [54, 57]. Furthermore, GPER agonist has been shown to improve cardiac function by inhibiting mitochondrial permeability transition pore (mPTP) opening and activating the AMPK signaling [37]. Moreover, GPER activation also ameliorates dyslipidemia by enhancing AMPK signaling [58]. Collectively, these data suggest that GPER may serve as a therapeutic target for metabolic disorders in postmenopausal women.

In the last part of this study, it was shown that induction of diabetes with a HFD led to a decrease in UCP2 level and oxidative stress, which were exacerbated in the concomitant presence of diabetes and menopause. In contrast, however, GPER stimulation was able to counteract these changes by increasing UCP2 and SOD and decreasing MDA. Evidence suggests that diabetes can accelerate cardiomyopathy through cardiac mitochondrial dysfunction and induction of oxidative stress [59]. Therefore, preventing excessive oxidative stress can improve heart function. UCP2 is a member of inner mitochondrial membrane proteins that plays important roles in regulating fatty acid oxidation, mitochondrial biogenesis, and oxidative stress, and its reduction, which occurs in diabetes and obesity, is associated with increased oxidative stress and cardiac dysfunction [60]. Also, increased levels of peroxidative lipid production, such as MDA, and decreased SOD activity in both diabetic hearts [61] and cardiomyocytes in high-glucose culture media [62] have been observed. The decrease in UCP2 and the increase in oxidative stress observed in menopausal conditions in this study are consistent with other studies [41, 63, 64]. Rogers et al. [65] demonstrated that ovariectomy leads to a decrease in UCP2, which in turn reduces the oxidative capacity of fatty acids. This reduction in oxidative capacity results in an increase in oxidative stress and harmful cardiac changes. Ovarian hormones have major effects on metabolic status of female mammals through central and environmental mechanisms that control energy consumption, and by acting on the AMPK-mTOR signaling pathway, improve the activity of antioxidant enzymes and attenuate the production of oxidative stress [66]. G-1 has been reported to reduce oxidative stress in the brains of older mice by increasing UCP2. GPER agonists also improve brain function by increasing mitochondrial biogenesis and function, which decreases during menopause [64]. Total antioxidant capacity and SOD are increased by treatment with G-1 in older OVX mice, and G-1 prevents vascular damage by altering oxidative and antioxidant parameters [67].

## Conclusions

**Fig 6:**
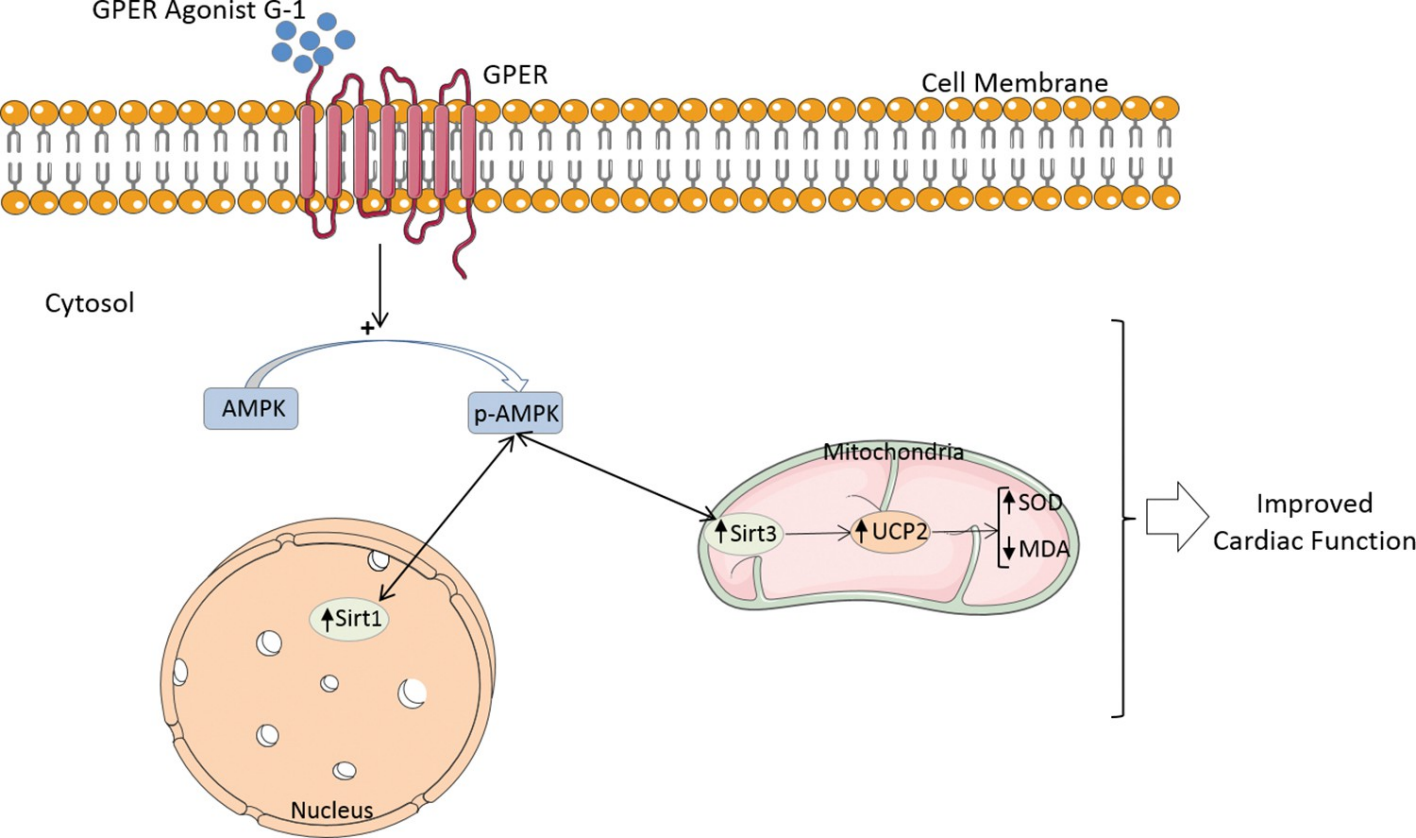
Schematic representation of the proposed underlying mechanism by which the GPER agonist G-1 exerts a cardioprotective effect via the Sirt1/3-AMPK-UCP2 pathway.

In conclusion, T2D in female animals leads to cardiac dysfunction through decrease in sirtuins, p-AMPK, UCP2, and SOD and an increase in MDA in the heart. Furthermore, E2 deficiency (menopausal model) exacerbates these changes. However, treatment with a GPER agonist, the G-1, may at least partially counteract the potentiating effects of menopause. Taken together, our results provide evidence to conclude that elevation of cardiac Sirt1/3, p-AMPK, and UCP2 levels by G-1 may improve mitochondrial function in menopausal diabetic female hearts, which may reverse oxidative stress and thus potentially improve cardiac metabolic status and function (Fig 6). In future translational studies in women, we will examine whether the beneficial effect of G-1 on cardiac treatment in postmenopausal diabetic women can also be conclusively demonstrated.

## Supporting information

S1 Raw images (PDF)
