# Supplementary material for: GPER activation attenuates cardiac dysfunction by upregulating the SIRT1/3-AMPK-UCP2 pathway in postmenopausal diabetic rats
Source: PLoS One. 2023 Dec 22;18(12):e0293630. doi: 10.1371/journal.pone.0293630 (PMC10745199; doi:10.1371/journal.pone.0293630)

Fig 3 (A). Sirt1, 110 KDa

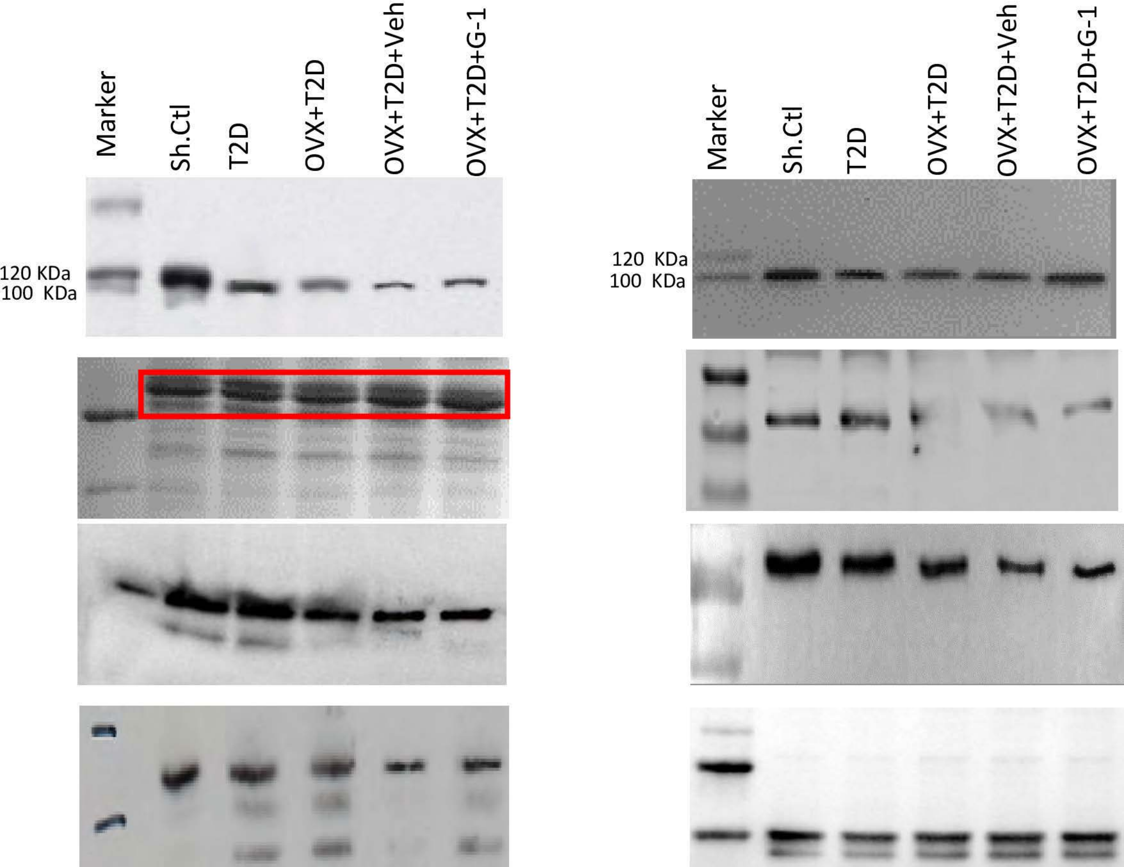

Fig 3 (B). Sirt2, 43 KDa

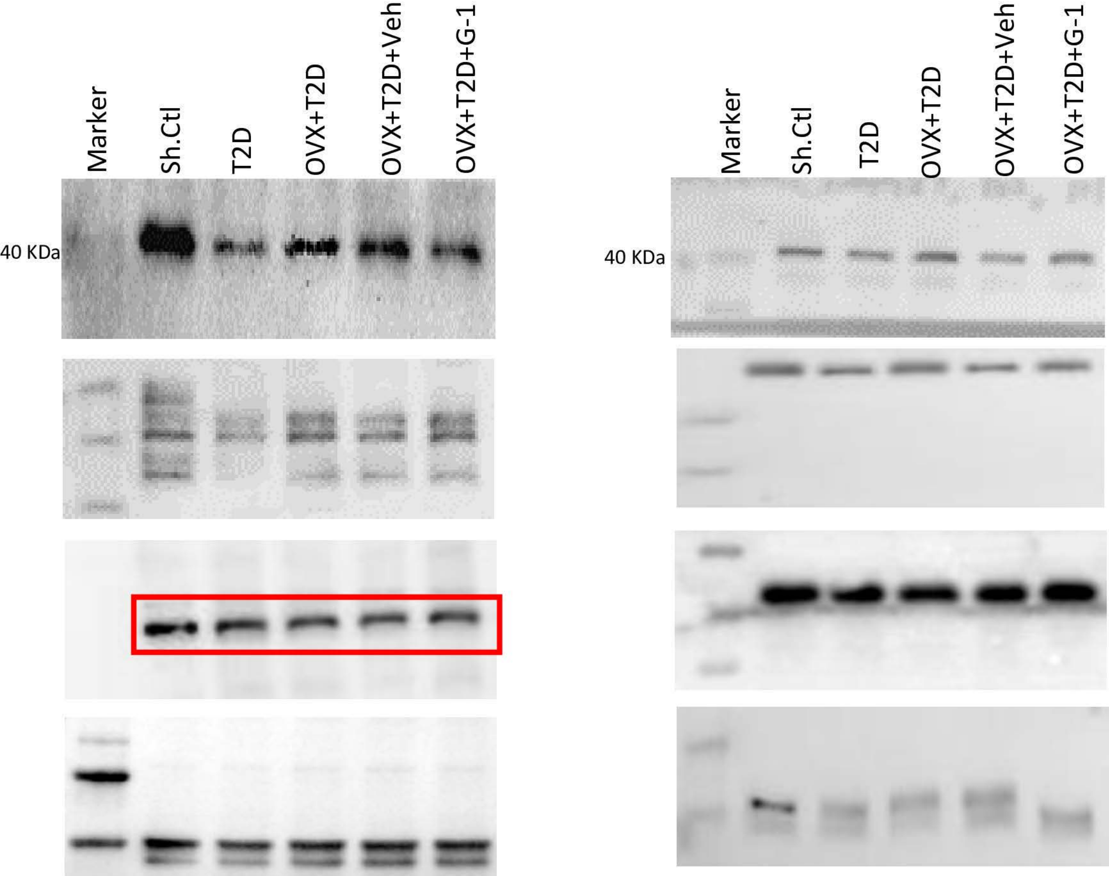

Fig 3 (C). Sirt3, 28 KDa

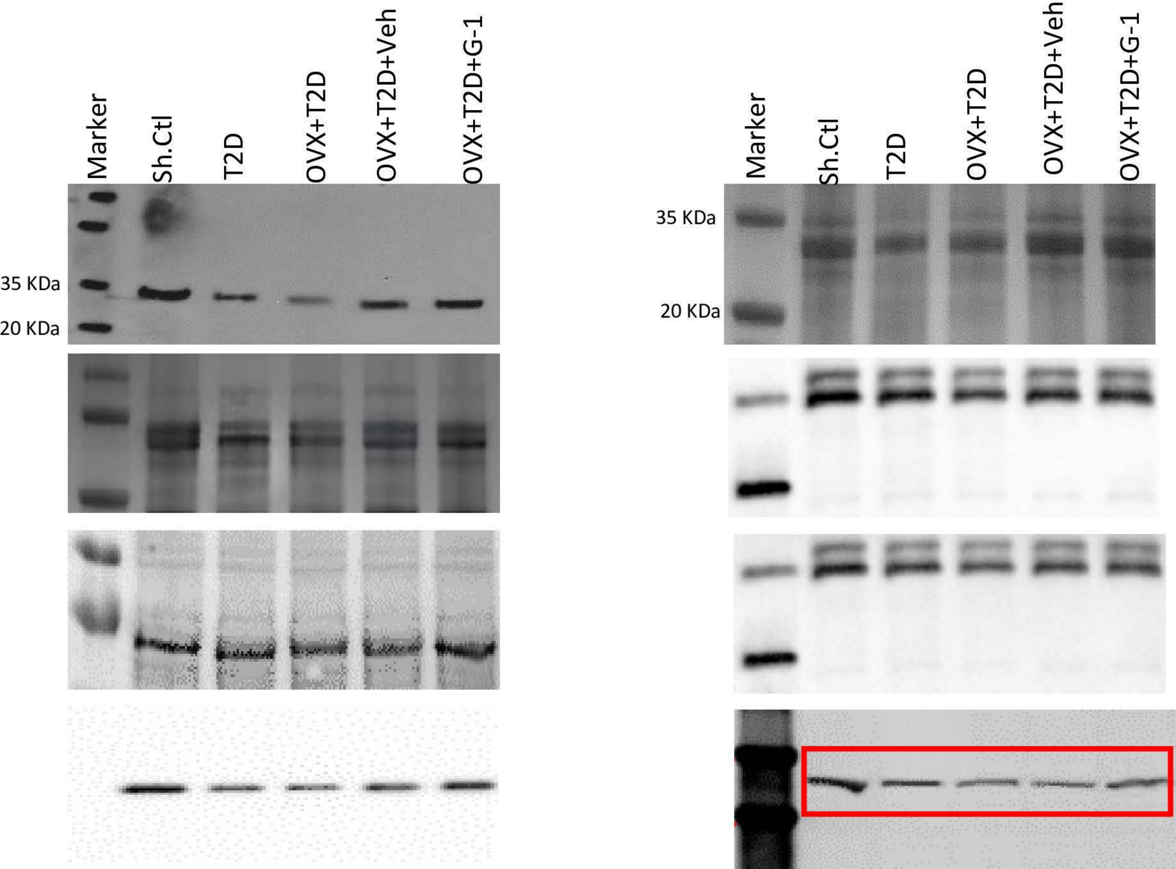

Fig 3 (D). Sirt6, 40 KDa

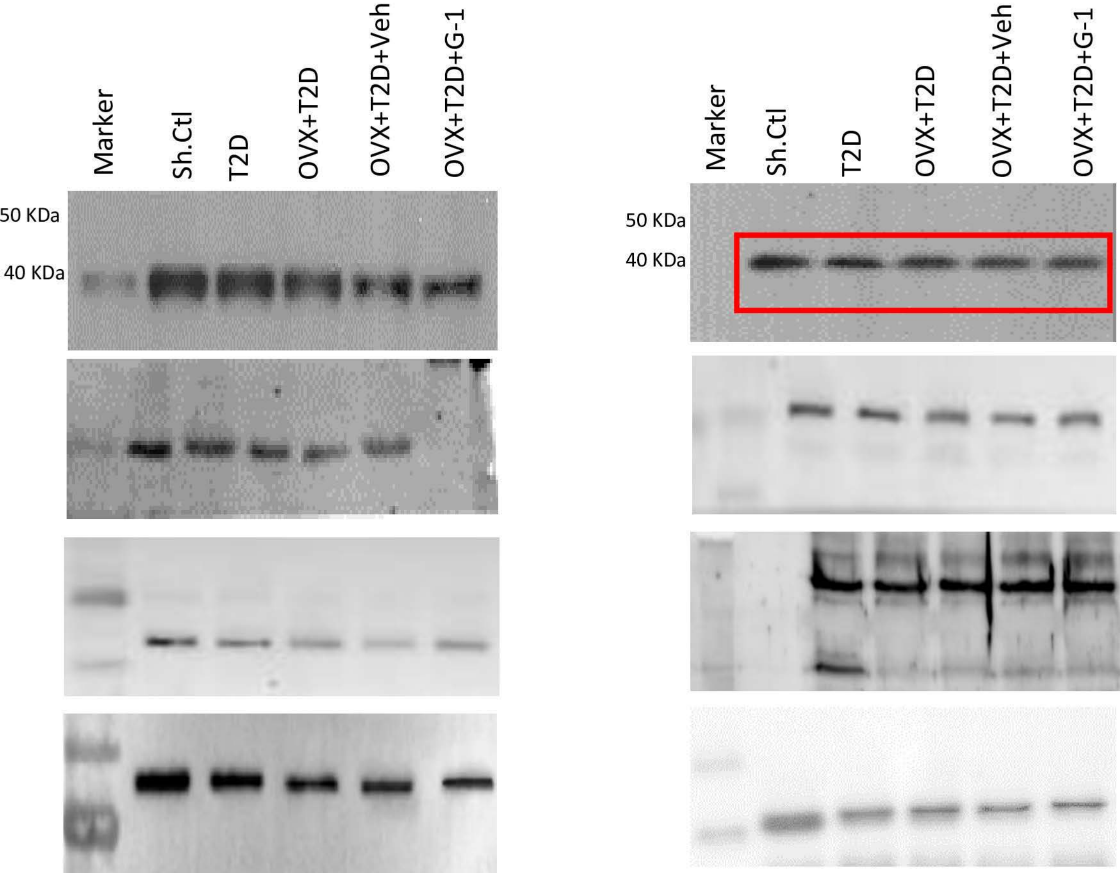

Fig 4. AMPK, 62KDa

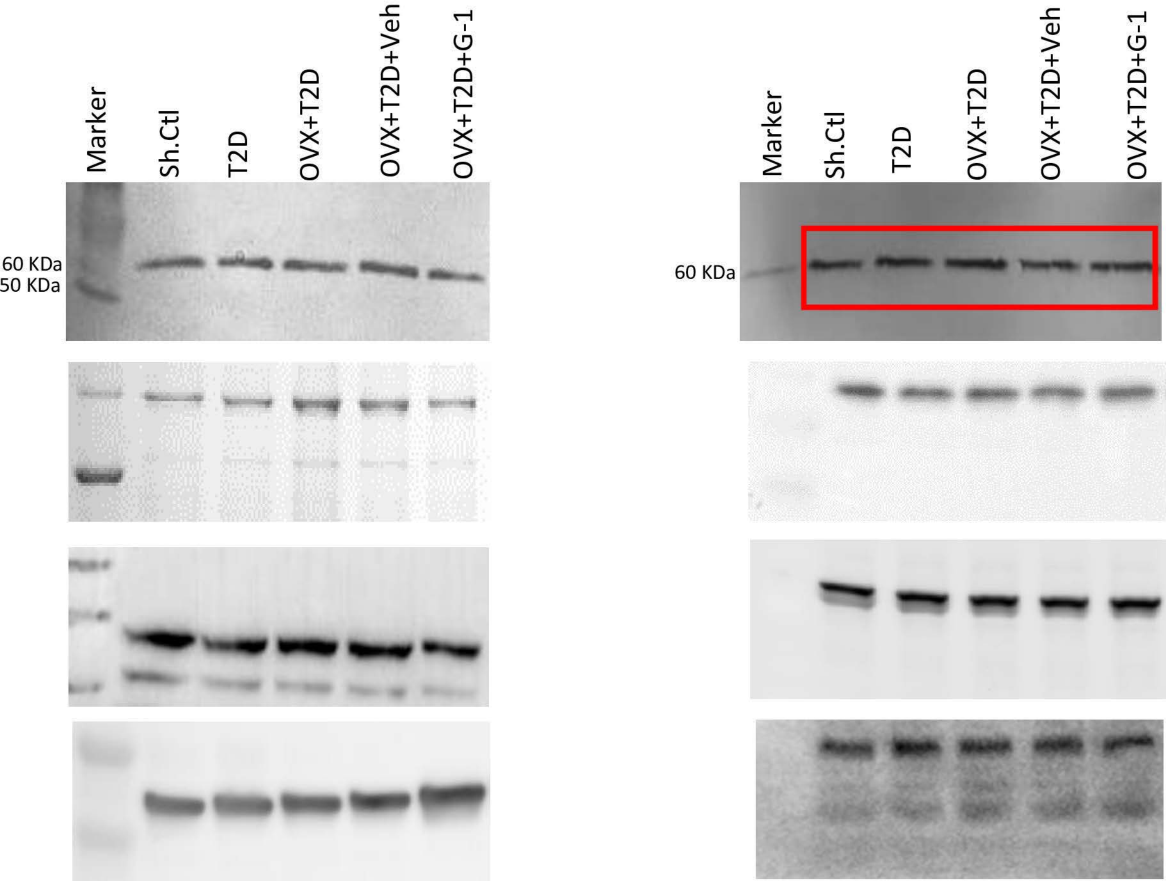

Fig 4. p-AMPK, 62KDa

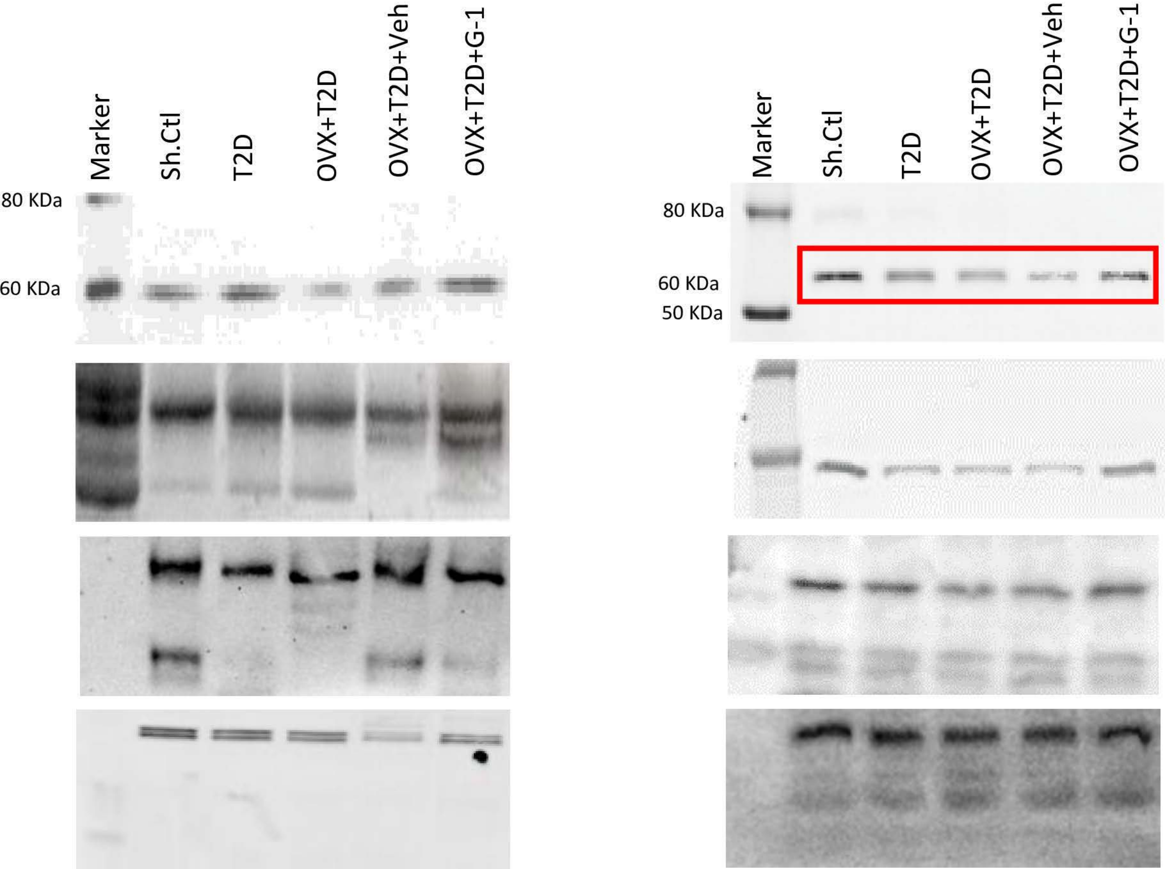

Fig 5 (A). UCP2, 35 KDa

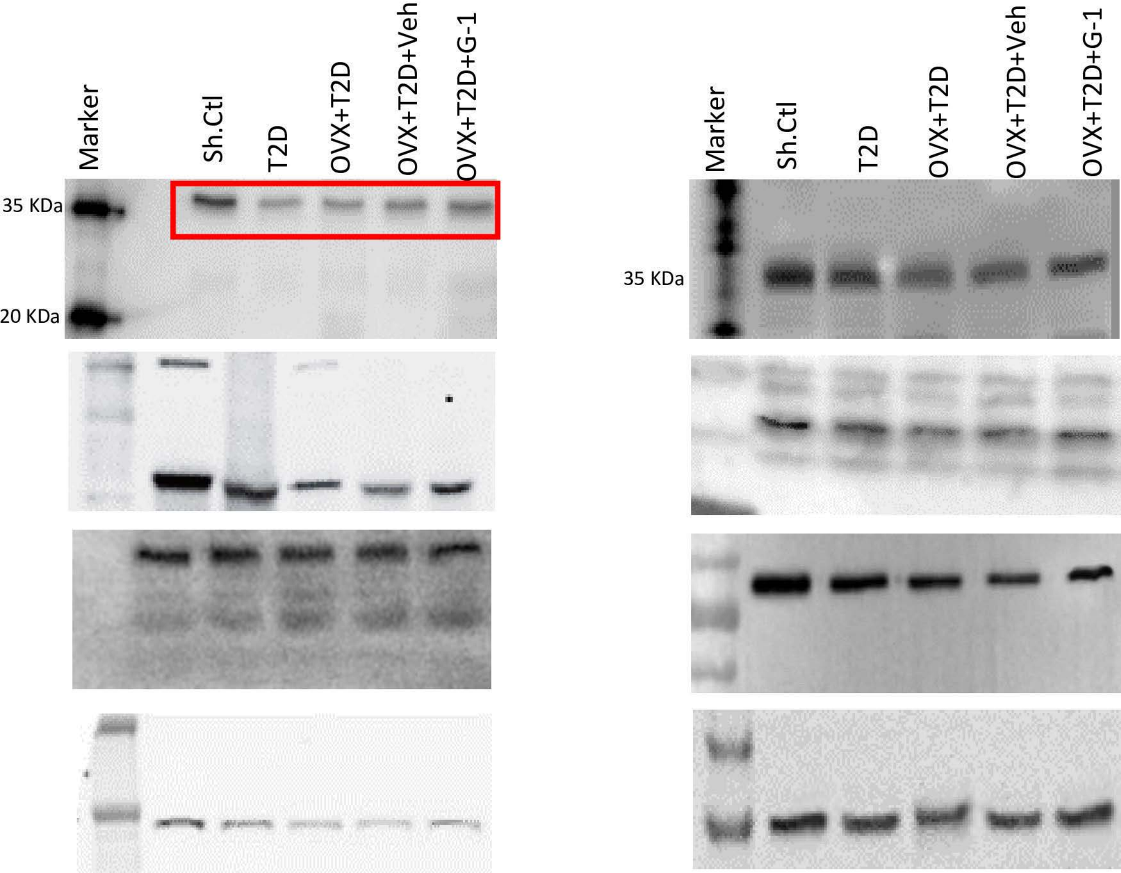

β-Actin. 43 KDa

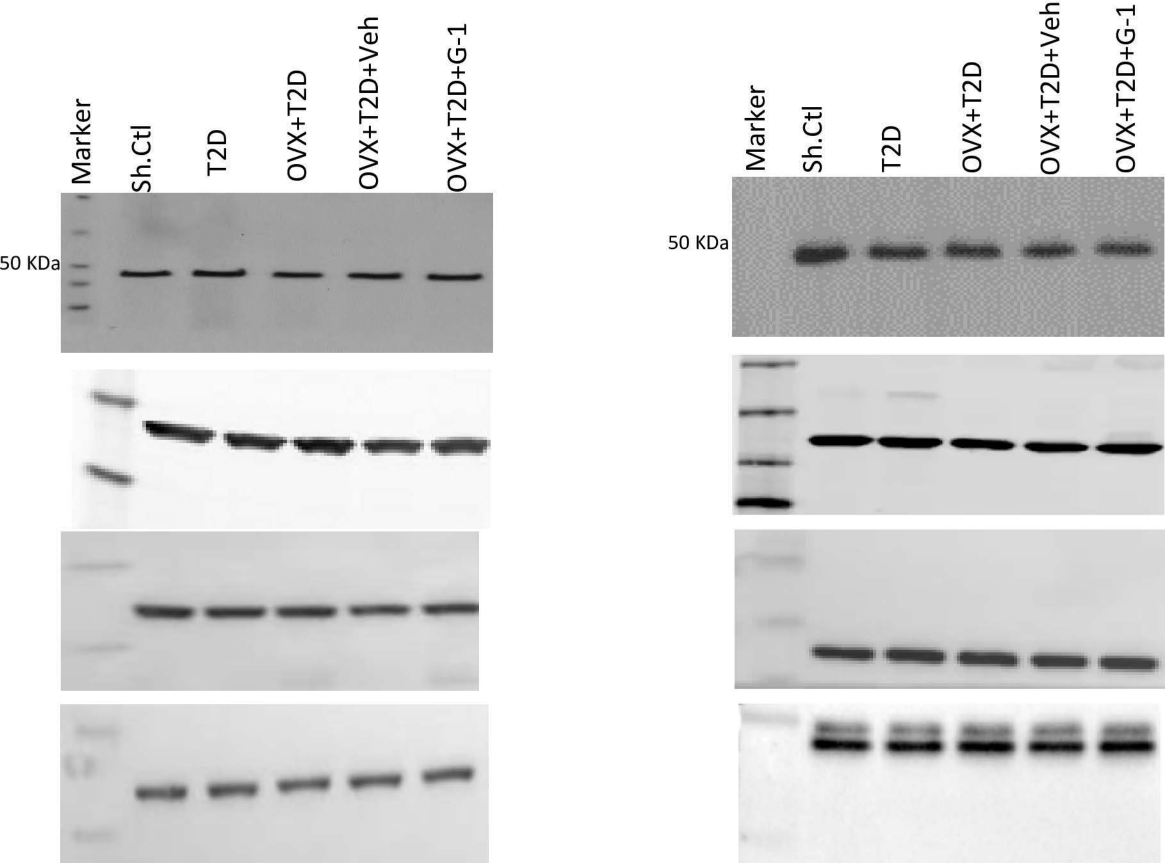

Supplement: S1 Raw images — (PDF) [file pone.0293630.s001.pdf]
